# Tryptophan, Kynurenine and Kynurenic Acid Concentrations in Milk and Serum of Dairy Cows with *Prototheca* Mastitis

**DOI:** 10.3390/ani11123608

**Published:** 2021-12-20

**Authors:** Mariola Bochniarz, Tomasz Piech, Tomasz Kocki, Mateusz Iskra, Henryk Krukowski, Tomasz Jagielski

**Affiliations:** 1 Department and Clinic of Animal Reproduction, Faculty of Veterinary Medicine, University of Life Sciences, Gleboka 30, 21-612 Lublin, Poland; mariola.bochniarz@up.lublin.pl (M.B.); tomasz.piech@up.lublin.pl (T.P.); 2 Department of Experimental and Clinical Pharmacology, Medical University of Lublin, Jaczewskiego 8b, 20-090 Lublin, Poland; tomasz.kocki@umlub.pl; 3 Department of Medical Microbiology, Institute of Microbiology, Faculty of Biology, University of Warsaw, I. Miecznikowa 1, 02-096 Warsaw, Poland; m.iskra@biol.uw.edu.pl; 4 Department of Animal and Environmental Hygiene, University of Life Sciences in Lublin, Akademicka 13, 20-950 Lublin, Poland; henryk.krukowski@up.lublin.pl

**Keywords:** cows, mastitis, *Prototheca* spp., tryptophan, kynurenine, IDO

## Abstract

**Simple Summary:**

Bovine mastitis continues to be a leading cause of heavy economic losses in the dairy industry worldwide. Among a broad spectrum of infectious agents implicated in this pathology are unicellular, achlorophyllous microalgae of the genus *Prototheca*. The prevalence of mastitis due to *Prototheca* algae is currently increasing. The aim of this work was to explore a possible association of the kynurenine pathway of tryptophan metabolism with *Prototheca* mastitis. The authors were eager to know whether metabolites of tryptophan degradation can be used as markers of the protothecal bovine mastitis. Both tryptophan and its metabolite kynurenine occurred at a significantly lower level in the milk of cows with mastitis compared to healthy animals. Whereas the activity of indoleamine 2,3-dioxygenase, a key enzyme of tryptophan catabolism, was significantly higher in milk of mastitic cows compared to control animals. It was thus concluded that low values of tryptophan and kynurenine concentrations or elevated indoleamine 2,3-dioxygenase activity in milk samples can be used as markers of mastitis due to *Prototheca* spp.

**Abstract:**

The aim of this work was to investigate serum and milk levels of tryptophan (TRP), kynurenine (KYN), and kynurenic acid (KYNA), as well as the activity of indoleamine 2,3-dioxygenase (IDO) in cows with mastitis due to *Prototheca* algae. The study was prompted by previous research showing a link between the KYN pathway of TRP metabolism and bovine mastitis of bacterial etiology. The study was carried out over a 2-year period (2018–2019) and included quarter milk and serum samples collected from six dairy herds in Poland. The samples were obtained from healthy cows and cows with *Prototheca* mastitis of either clinical and subclinical manifestation, as determined upon direct measurement of the somatic cell count or indirectly by performing a California Mastitis Test on suspected quarters. Both TRP and KYN concentrations were significantly lower in milk of mastitic cows compared to healthy animals (0.8 vs. 8.72 µM, *p* = 0.001; 0.07 vs. 0.32 µM, *p* = 0.001, respectively). The difference in TRP and KYN concentrations in the sera of the two animal groups was much less pronounced (25.55 vs. 27.57 µM, 3.03 vs. 3.56 nM, respectively). The concentration of KYNA was almost at the same level in milk (1.73 vs. 1.70 nM) and in serum (80.47 vs. 75.48 nM) of both mastitic and healthy cows. The data showed that the level of TRP and its metabolites in serum was conspicuously higher compared to milk in all cows under the study. The activity of IDO was significantly higher in milk of cows with *Prototheca* mastitis compared to healthy animals (71.4 vs. 40.86, *p* < 0.05), while in serum it was pretty much the same (135.94 vs. 124.98, *p* > 0.05). The IDO activity differed significantly between serum and milk both for mastitic (135.94 vs. 71.4, *p* < 0.05) and healthy cows (124.98 vs. 40.86, *p* < 0.001). In conclusion, low values of TRP and KYN concentrations or elevated IDO activity in milk samples might be used as markers of mastitis due to infectious causes, including *Prototheca* spp.

## 1. Introduction

Tryptophan (TRP) belongs to the group of nutritionally essential, exogenous amino acids and plays many important roles in animals and humans [1,2,3]. The activity of TRP is multidirectional. One of its pivotal functions is as a substrate for protein synthesis. Supplementation with TRP clearly enhances feed utilization, and thus boosts livestock productivity [4,5,6]. TRP-deficient diet leads to depressed body weight gain, lowered food intake, and an overall metabolic disturbance, which makes an animal more prone to infections and other ailments, thus increasing morbidity and mortality among the herds [3,7,8,9]. TRP has also been shown to counteract the stress response in animals, serving as a precursor of such important neuroactive molecules as serotonin and melatonin [10,11].

TRP undergoes several metabolic pathways occurring mainly in liver [2,5]. In animals, as in humans, only around 1% of TRP is used for protein synthesis. Likewise, a very small portion (<5%) of the amino acid is hydrolysed to 5-hydroxytryptophan, leading to the formation of serotonin, and subsequently melatonin, responsible for regulating mental health and circadian rhythms, respectively [12,13]. In contrast, more than 90% of TRP is metabolized via the kynurenine pathway (KP), with two enzymes, namely indoleamine 2,3-dioxygenase (IDO) and TRP 2,3-dioxygenase (TDO), catalyzing the first step of the pathway [14,15]. A key metabolite in KP is l-kynurenine (l-KYN, further in the text referred to as KYN), which is a substrate for further metabolic transformations yielding a myriad of biologically active compounds. The KP metabolites, collectively referred to as kynurenines, have been demonstrated to be involved in many physiological and pathological processes [16]. Kynurenines have been shown to induce anti-inflammatory responses in the course of various infections, including bovine mastitis [15,17,18,19,20].

Mastitis is a multi-factorial disease, in which the pathogen, the host, and the environment are at a constant interplay with each other, determining the type and severity of the condition. Unicellular, achlorophyllous microalgae of the genus *Prototheca* have been increasingly implicated in the etiology of bovine mastitis [21,22,23,24,25,26,27,28]. These microorganisms are ubiquitously distributed in nature, with a clear predilection for humid and organic-rich environments. Of 15 currently recognized species, only four have been reported as the causative agents of animal protothecosis, namely *P. bovis* (formerly *P. zopfii* gen. 2), *P. blaschkeae*, *P. ciferrii* (formerly *P. zopfii* gen. 1), and *P. wickerhamii*. [29]. The vast majority of *Prototheca* mastitis cases are due to *P. bovis*, but *P. blaschkeae* infections do also occur, even in an outbreak context [26,27,30,31,32].

Protothecal bovine mastitis typically occurs as a chronic disease, either symptomatic or clinically silent, with periods of flare-up and latency [22,33]. There are currently no standard treatment regimens for bovine mammary protothecosis. Upon laboratory drug susceptibility testing, the algae show high levels of resistance towards a variety of antimicrobial agents [34,35]. Although the disease is rarely life-threating, its persistence, drug resistance, and irreversible damage to the mammary gland tissue, leading to a decrease or complete loss of milk production, advocate culling as the only disease intervention strategy [26,27].

The aim of the study was to investigate serum and milk concentrations of TRP, KYN, and kynurenic acid (KYNA), as well as to determine the IDO activity in cows with *Prototheca* mastitis and in healthy animals. The authors of this study were eager to know if and to what extent *Prototheca* mastitis affects KP, and whether the KP metabolites can be used as markers of this pathology.

The experiments reported here were prompted by our previous study which demonstrated a link between the kynurenine pathway of TRP metabolism and bovine mastitis of staphylococcal etiology [20].

## 2. Materials and Methods

### 2.1. Dairy Farms and Animals

The study was conducted over a two-year period (2018–2019) and included quarter milk samples (QMS) collected from 6 dairy herds (of 367 cows in total) in Eastern Poland (Lublin and Podlasie provinces).

The Holstein–Friesian cows were housed in brick-built, free-stall barns, with an average of 6.5 sq. m. total space per cow and a minimum window-to-floor ratio of 1:15. The barns were naturally ventilated through adjustable sidewall and roof air inlets. The floor of the barns was covered with straw and the manure was removed manually or with mechanical separators at least once a day.

On all farms under the study, milking was done twice daily in a double-rowed milking parlour or with a milking pipeline. Before milking, the udders were washed with clean, warm water and dried with a separate cloth, and the teats were dipped in a disinfectant, typically an iodine-containing solution. The same solution was used for post-milking teat disinfection.

Winter feeding was based on maize silage, grass-hay silage, meadow hay, and individually formulated grain mixtures. The animals also received concentrated feed, as well as vitamin and mineral supplements. In the summer period, cows fed on pastures, but were also offered green forage and, in a smaller amount than in winter, maize silage, concentrates, and vitamin and mineral supplements. Feed was delivered to the pens using a feed truck. Water was available ad libitum from automatic drinking troughs.

The general health of the dairy herds was monitored through a scheduled routine and veterinary visits. For all breeding, artificial insemination was used.

### 2.2. Sampling Procedures

The quarter milk samples were collected from cows suspected of having mastitis, based on the veterinary clinical examination as per the results of the California Mastitis Test (CMT; Mastirapid, Vetoquinol Biowet, Poland). Scoring of the CMT results was performed according to Quinn et al. (2002) [36]. CMT-positive QMS were examined for microbiological growth and were tested for somatic cell count (SCC), essentially as described elsewhere [26]. The SCC threshold for defining mastitis was 2 × 10^5^ cells mL^−1^. Clinical (CM) and subclinical (SCM) mastitis were defined as previously reported [26].

Milk sample collection procedures, culturing methods, and interpretative criteria of microbial growth were described essentially elsewhere [26]. Colonies suspected to be *Protothec* a spp. were subcultured on Sabouraud dextrose agar (SDA, Difco Laboratories, Detroit, MI, USA) and subjected to species identification, which included both phenotypic (morphological and auxanographic assays) and molecular methods, with the latter carried out as a PCR restriction-enzyme analysis (PCR-REA) assay of the partial *CYTB* gene [37].

### 2.3. Study Group

A total of 20 dairy cows were included as the study group. Comprised within this number were 10 cows identified as having *Prototheca* mastitis and 10 healthy (control) cows, chosen randomly among the herds investigated (i.e., 2 cows from each of the four herds and single cows from another two herds). The healthy status of control cows was supported by a lack of any clinical signs of mastitis, negative bacteriological examination, and SCC level of <100,000 cells/mL. For all animals, the complete blood cell (CBC) tests (incl. red blood cells, white blood cells, platelets, haemoglobin, and haematocrit) were performed using Scil ABC+ Vet Animal Hematology Analyzer (Horiba, Kyoto, Japan). The results of the CBC tests are provided in the Supplementary Table S1.

### 2.4. Measurements of TRP and Its Metabolites Concentrations in Milk and Serum

TRP, KYN, and KYNA were analyzed by high-performance liquid chromatography (HPLC), according to the protocol of Zhao et al. (2010), with minor adjustments [38]. Briefly, to each milk and serum sample, 6% HClO_4_ was added and the sample was centrifuged at 12,000× *g* for 30 min at 4 °C. The resulting supernatant was used for HPLC with the Dionex P680 Pump, UltiMate 3000 Autosampler with column compartment (Sunnyvale, CA, USA). Agilent HC-C18(2) column (250 × 4.6 mm i.d.; 5-µm particle size) was used coupled to RS Variable Wavelength UltiMate 3000 Detector (Dionex, Sunnyvale, CA, USA) set at wavelengths 250 nm for TRP, 365 nm for KYN, and RF 2000 Fluorescence Detector (Dionex) set at excitation wavelength of 348 nm and emission at 398 nm for KYNA determination. The mobile phase was a mixture of 20-mM NaAc, 5-mM ZnAc_2_, and 4% acetonitrile for which the flow rate was 1.0 mL/min. Control of the HPLC system and data collection were performed with the Chromeleon software (Dionex).

A full description and validation of the method is provided in the paper, by Zhao et al. (2010) [38].

IDO activity was calculated as a ratio by dividing the content of KYN (in µM multiplied by 1000) by that of TRP (in µM) [39].

### 2.5. Statistical Analysis

Statistical methods were used to compare concentrations of TRP, KYN, and KYNA, as well as IDO activities in serum and milk of healthy cows and cows with *Prototheca* mastitis. Statistical parameters included the minimum and maximum values, median, mean, and standard deviation (SD). The Mann–Whitney test was applied for comparison between these parameters for two independent trials, since the Shapiro–Wilk test showed a non-normal distribution of data. A value of *p* < 0.05 was considered significant. All calculations were performed using the statistical package of IBM SPSS Statistics for Windows, version 24.0 (IBM, Armonk, NY, USA).

## 3. Results

Out of a total of 121 cows examined, 48 were CMT-positive. Among these, 4 (8.3%) did not show any microbial growth upon culture, whereas the remaining 44 (91.7%) were identified as having mastitis due to either bacteria (31 or 70.5%; coagulase-negative *Staphylococcus* spp. (CNS)—15 or 34.1%; *Staphylococcus aureus*—2 or 4.6%; *Streptococcus agalactiae*—1 or 2.3%; *Streptococcus dysgalactiae*—4 or 9.1%; *Streptococcus uberis*—6 or 13.6%; *Escherichia coli*—3 or 6.8%) yeasts (*Candida* spp.—3 or 6.8%) or *Prototheca* spp. (10; 22.7%). In all *Prototheca* mastitis cases, *P. bovis* was the etiological agent. These cows came from all 6 farms under the study; four cows were single cases (a case per herd), whereas the other six represented clustered cases (i.e., two cows in one herd, and four cows in another herd were affected). Eight cows had subclinical mastitis, and the remaining two presented with a clinical disease. The SCC values in milk samples of these cows fell within a wide range of 406,000–10,000,000 cells/mL.

From all sick animals, 10 serum samples and 15 milk samples were collected (in 6 cows, *Prototheca* spp. were cultured from one quarter only, in 3 cows—from two quarters, and in one cow—from three quarters). One milk sample was heavily flaky, making assessment of TRP and kynurenine levels impossible. Thus, the results were obtained and presented for 14 milk samples.

TRP, KYN, and KYNA Concentrations in Milk and Serum of Cows with Prototheca Mastitis and Healthy Animals

**Figure 1:**
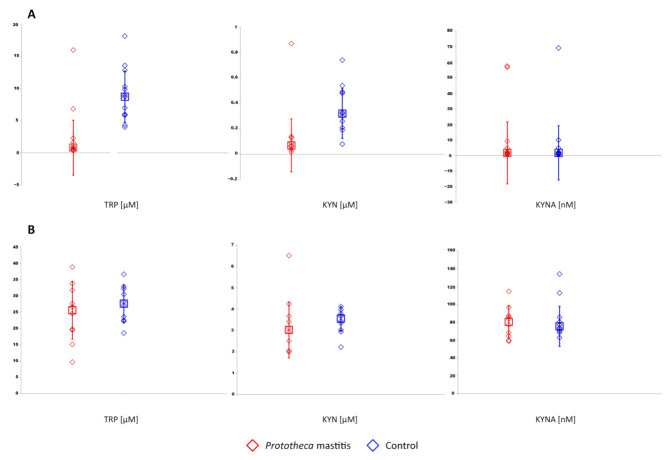
TRP, KYN, and KYNA concentrations in milk (**A**) and serum (**B**) of cows with *Prototheca* mastitis and control animals. The median and standard deviation are represented as squares and vertical lines, respectively.

TRP and KYN concentrations were significantly lower in the milk of cows with *Prototheca* mastitis compared to healthy animals (0.8 vs. 8.72 µM, *p* = 0.001; 0.07 vs. 0.32 µM, *p* = 0.001, respectively) (Figure 1A). However, there was no statistically significant difference in TRP and KYN concentrations in sera of animals from the two groups (25.55 vs. 27.57 µM, 3.03 vs. 3.56 nM, respectively) (Figure 1B). The concentration of KYNA was almost at the same level in milk (1.73 vs. 1.70 nM) and in serum (80.47 vs. 75.48 nM) of both mastitic and healthy cows (Figure 1).

The data show that the levels of TRP and its metabolites in serum were conspicuously higher compared to milk in all cows under the study. Serum TRP levels in healthy cows ranged from 18.56 to 36.55 µM (median 27.57 µM) and were several times higher than in the milk of these animals (3.99–18.11 µM, median 8.72 µM, *p* = 0.001). Likewise, in mastitic cows, a significant difference in median TRP concentrations in serum and milk was noted (25.55 vs. 0.8 µM, *p* < 0.001). The same observation was made for the levels of TRP metabolites, i.e., KYN and KYNA. Both in the group of healthy and diseased cows, the levels of KYN (3.56 vs. 0.32 µM, *p* < 0.001; 3.03 vs. 0.07, *p* < 0.001, respectively) and KYNA (75.48 vs. 1.70 nM, *p* < 0.001; 80.47 vs. 1.73 nM, *p* < 0.001, respectively) were significantly higher in serum than in milk.

**Figure 2:**
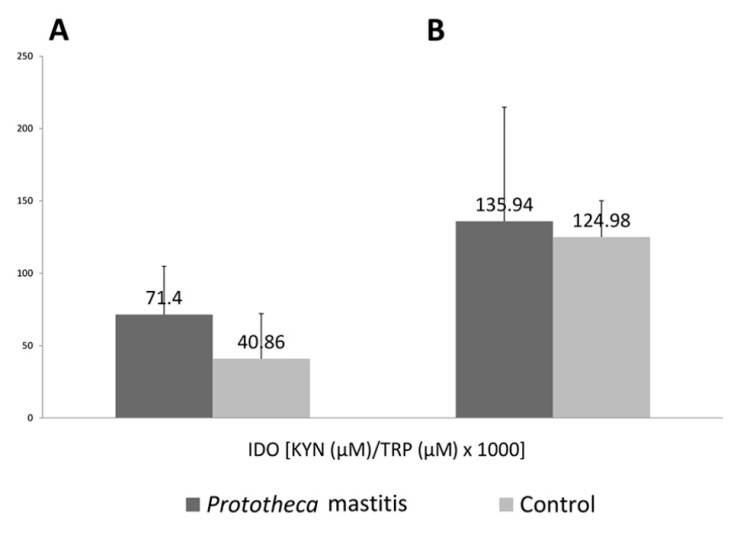
IDO activity in milk (**A**) and serum (**B**) of cows with *Prototheca* mastitis and control animals. Numbers above the bars are median values, while vertical lines represent standard deviation.

IDO activity, determined as KYN/TRP ratio, was significantly higher in the milk of cows with mastitis compared to healthy cows (71.4 vs. 40.86, *p* < 0.05). Whereas, IDO activity in sera of cows of the two groups was almost at the same level (135.94 vs. 124.98, *p* > 0.05). The IDO activity differed significantly between serum and milk both for mastitic (135.94 vs. 71.4, *p* < 0.05) and healthy cows (124.98 vs. 40.86, *p* < 0.001). (Figure 2).

## 4. Discussion

This study investigated, for the first time, the concentrations of TRP, KYN, KYNA, and the activity of IDO in serum and milk of cows with *Prototheca* mastitis.

One of the processes regulating the course and severity of inflammation is the activation of TRP metabolism via the KP [14,17,40]. While IDO plays only a minor role in TRP metabolism under physiological conditions, the KP pathway is strongly activated in response to cytokines secreted during inflammation [15,40,41].

In our study, a significant increase in IDO activity in milk was observed during the *Prototheca* mastitis infection. The IDO activity in *Prototheca*-contaminated milk was nearly two-fold that in milk from healthy cows. At the same time, the serum IDO activities were quite the same in both mastitic and control animals. This suggests that the inflammatory process induced by the algae occurs locally in the udder and does not trigger a systemic immune response. This is further supported by the lack of any general symptoms in cows with *Prototheca* mastitis. Significantly, the high IDO activity in milk of mastitis cows correlated with a significant increase in their milk SCCs. The mean SCC value calculated for milk from *Prototheca*-infected udders was 4,689,933 cells, mL^−1^, being over 40-fold higher compared to a threshold limit for milk acceptance, typically set at <100,000 cells/mL [42]. This indicates increased migration of leukocytes, mainly neutrophils and macrophages to the inflammatory focus within the mammary gland.

According to the literature, the expression of IDO in the immune response cells, and macrophages in particular, is induced upon different stimuli, including pro-inflammatory cytokines [3,43]. Interferon γ (IFN-ɣ) is believed to be the most potent activator of this enzyme [44]. Consequently, stimulation of IDO activity shifts TRP metabolism towards the KP, leading to KYN production, and thereby significantly reducing the concentration of available TRP. This, as repeatedly evidenced, inhibits growth of many microbial pathogens including intracellular parasites (*Toxoplasma gondii*, *Plasmodium* sp.), bacteria (*Pseudomonas aeruginosa, Chlamydia*, *Rickettsia*, *Streptococcus* sp., *Staphylococcus* sp.), and viruses (*Herpes simplex virus*) [40,45,46,47,48,49].

In our study, a clear relationship was found between IDO activation and TRP levels. Along with a significant increase of the IDO activity in milk, over a 10-fold lower level of TRP was recorded in the milk of mastitis cows compared to the control group. Still, at the serum level, no changes in the TRP concentrations were noted between healthy and diseased cows, since in the latter no IDO activation occurred. One may point to generally much higher values of TRP (and its metabolites) concentrations in sera than in milk samples. This is what was observed in previous studies. The TRP levels in serum consistently surpassed those in milk, irrespective of the physiological state of the udder [20,50].

The difference between the serum and milk level of TRP is plausibly due to an extensive use of this (and other) amino acid for milk protein synthesis in the mammary gland tissue, and the production of gluconeogenic and ketogenic precursors [50]. Captivatingly, the TRP concentration in milk of healthy cows was about 30% of its level in serum, while in the *Prototheca* mastitis cows, the level of TRP in milk was merely 3% of that found in serum.

Finally, the results of this study indicate that in the course of mastitis and KP activation, TRP, or at least some of its amount, can be metabolized in ways other than the normal healthy conditions. This is illustrated by the milk levels of KYN in mastitis and control cows. Among the latter, the KYN concentrations were 4-fold higher compared to mastitis animals. It is thus possible that KYN, which is the first product of increased TRP catabolism, undergoes an accelerated transformation (into more downstream metabolites) in the KP. Depending on the degree of cell specialization, KYN metabolism occurs through three branches, leading to the production of either 3-hydroxy-kynurenine (3-OH-KYN), anthranilic acid (AA) or KYNA [17,49,51]. In this study, the KYNA concentration in milk of mastitis cows equaled that in healthy animals. Altogether, it seems likely that KYN was metabolized via an alternative to KYNA-yielding catabolic pathway.

A limitation of the study was a relatively small sample size. Despite being on the rise, *Prototheca* mastitis is not as common as bacterial udder infections. Still, providing more *Prototheca*-positive milk samples would significantly improve the accuracy of the analysis and strengthen our conclusions.

In summary, the present work adds to very scanty research on *Prototheca* mastitis pathogenesis, providing details of one of the aspects of cellular response to *Prototheca*-induced inflammation of the mammary gland tissue. A significant increase of IDO activity in the milk of mastitis cows was closely related to a decrease of TRP and KYN concentrations. These findings may have important implications for diagnosing *Prototheca* mastitis, especially in cases without general symptoms or changes in milk or the mammary gland. Here, the activation of the KYN pathway and its metabolites might be used as markers of mastitis due to *Prototheca* spp. The mechanism might be the same as with other (bacterial) mastitis pathogens. Upon infection, a local (intramammary) immunological response develops, leading to an increased number of leukocytes and macrophages at the site of inflammation. These cells release excessively IDO-inducing pro-inflammatory cytokines, which in turn diverts TRP towards the kynurenine pathway.

## Data Availability

None of the data were deposited in an official repository.

## Supplementary Materials

The following are available online at https://www.mdpi.com/article/10.3390/ani11123608/s1. Table S1: Complete blood test cel results for both cows with *Prototheca* mastitis and healthy (control) animals.
