# Supplementary material for: Tryptophan, Kynurenine and Kynurenic Acid Concentrations in Milk and Serum of Dairy Cows with Prototheca Mastitis
Source: Animals (Basel). 2021 Dec 20;11(12):3608. doi: 10.3390/ani11123608 (PMC8698139; doi:10.3390/ani11123608)
Supplement: Supplementary file 1 [file animals-11-03608-s001.zip › animals-1498824-supplementary.pdf]

Table S1. Complete blood test cel results for both cows with Prototheca mastitis and healthy (control) animals.

	WBC [10^3/mL]	RBC [10^6/mL]	HT [%]	HGB [g/dL]	PLT [10^3/mL]	LYM [%]	GRAN [%]	EOS [%]	MON [%]	BAS [%]
Prototheca mastitis										
1.	8.0	6.44	30.2	10.6	364	50	39	7	4	0
2	11.1	8.98	40	13.4	401	61	31	4	3	1
3	5.2	5.58	25.7	8.8	480	53	33	8	5	1
4	6.8	6.74	34.9	11.3	772	56	29	13	0	2
5	5.1	5.66	24.3	8.1	553	52	32	15	1	0
6	5.0	5.30	24.2	8.2	648	37	15	44	3	1
7	7.6	5.71	26.6	8.9	425	43	40	14	2	1
8	5.2	5.63	27.6	9.5	549	40	24	35	1	0
9	5.9	4.83	22.6	7.4	475	66	23	11	0	0
10	7.1	7.9	31.7	11.2	276	38	30	27	5	0
Mean	**6.7**	**6.3**	**28.8**	**9.7**	**494.3**	**49.6**	**29.6**	**17.8**	**2.4**	**0.6**
Control										
1	5.0	5.78	30.3	10.1	391	52	35	6	6	1
2	6.3	6.17	25.8	8.7	630	45	44	7	3	1
3	5.8	6.90	32.5	10.6	358	57	35	3	5	0
4	8.8	7.03	27.2	8.7	624	55	33	10	2	0
5	7.3	6.02	24.9	8.3	488	58	34	6	1	1
6	5.9	6.13	28.8	9.6	375	44	40	13	1	2
7	5.7	6.73	31.7	10.4	249	62	28	4	5	1
8	5.9	7.43	33.5	10.9	387	55	29	3	12	1
9	8.0	5.02	21.5	7.2	620	55	35	9	1	0
10	7.6	6.33	26.8	8.9	407	72	18	7	3	0
Mean	**6.6**	**6.3**	**28.3**	**9.3**	**452.9**	**55.5**	**33.1**	**6.8**	**3.9**	**0.7**
